# Predictive value of preoperative inflammatory indexes for postoperative early recurrence of hepatitis B-related hepatocellular carcinoma

**DOI:** 10.3389/fonc.2023.1142168

**Published:** 2023-07-13

**Authors:** Guo Wenpei, Li Yuan, Li Liangbo, Mu Jingjun, Wang Bo, Niu Zhiqiang, Ning Yijie, Liu Lixin

**Affiliations:** ^1^ Department of Gastroenterology and Hepatology, The First Hospital of Shanxi Medical University, Taiyuan, China; ^2^ Department of Respiratory Medicine, Shanxi Province Cancer Hospital, Shanxi Hospital Affiliated to Cancer Hospital, Chinese Academy of Medical Sciences, Cancer Hospital Affiliated to Shanxi Medical University, Taiyuan, China; ^3^ Department of Stomatology, Chinese PLA General Hospital, Beijing, China; ^4^ Department of Urinary Surgery, Shanxi Province Cancer Hospital, Shanxi Hospital Affiliated to Cancer Hospital, Chinese Academy of Medical Sciences, Cancer Hospital Affiliated to Shanxi Medical University, Taiyuan, China; ^5^ Department of Pathology, Shanxi Province Cancer Hospital, The First Hospital of Shanxi Medical University, Taiyuan, China; ^6^ Department of Hepatobiliary Surgery, The First Hospital of Shanxi Medical University, Taiyuan, China; ^7^ Department of Neurosurgery, The First Hospital of Shanxi Medical University, Taiyuan, China; ^8^ Experimental Center of Science and Research, The First Hospital of Shanxi Medical University, Taiyuan, China; ^9^ Institute of Liver Diseases and Organ Transplantation, The First Hospital of Shanxi Medical University, Taiyuan, China

**Keywords:** inflammatory indexes, hepatocellular carcinoma, recurrence, predictive models, hepatitis B virus

## Abstract

**Objective:**

To investigate the predictive value of preoperative neutrophil-to-lymphocyte ratio (NLR), platelet-to-lymphocyte ratio (PLR), systemic inflammation response index (SIRI), and systemic immune inflammation index (SII) for early recurrence after liver resection in patients with hepatitis B-related hepatocellular carcinoma.

**Methods:**

A retrospective study was conducted on 162 patients who underwent hepatitis B-related hepatocellular carcinoma (HCC) resection between January 2013 and April 2016. The Youden index was utilized to calculate the optimal cut-off value. The Pearson Chi-square test was applied to analyze the relationship between inflammatory indexes and common clinical and pathological features. The Kaplan-Meier method and Log-Rank test were implemented to compare the recurrence-free survival rate within 2 years of the population. The Cox regression analysis was used to identify the risk factors for early postoperative recurrence.

**Results:**

The best cut-off values of SIRI, PLR, NLR and SII were 0.785, 86.421, 2.231 and 353.64, respectively. Tumor diameter, degree of tumor differentiation, vascular invasion, SIRI>0.785, PLR>86.421, NLR>2.231 and SII>353.64 were risk factors for early recurrence. Combining the above seven risk factors to construct a joint index, the AUC of the joint prediction model was 0.804. The areas under the ROC curves of SIRI, PLR, NLR, and SII were 0.659, 0.725, 0.680, and 0.723, respectively. There was no significant difference in the predictive ability between the single inflammatory index models, but the predictive performance of the joint prediction model was significantly higher than that of the single inflammatory index models. The patients with lower SIRI, PLR, NLR, SII and joint index value had longer recurrence-free survival within 2 years.

**Conclusion:**

The joint index CIP, constructed by combining preoperative SIRI, PLR, NLP and SII with pathological features, can better predict the early recurrence of HBV-related HCC patients after surgery, which is beneficial in identifying high-risk patients and assisting clinicians to make better clinical choices.

## 1 Introduction

Primary liver cancer is one of the most malignant and influential cancers in the world. Its incidence is increasing year by year, but the treatment methods are extremely limited (1). Hepatocellular carcinoma (HCC) is the most common pathological type of primary liver cancer, accounting for about 75%-90% of all cases. The causes of HCC include hepatitis B virus (HBV) infection, hepatitis C virus infection, aflatoxin infection, alcohol consumption, and non-alcoholic fatty liver disease which has attracted much attention in recent years (2, 3). In China, HBV-related HCC accounts for the largest proportion. Although the infection rate of HBV is decreasing with the application of antiviral drugs, there is still a considerable base of HBV-related HCC in China (4).

The treatment methods for early and middle stage HCC include radiofrequency ablation, liver transplantation, and hepatectomy, among which hepatectomy is the most widely used. Tumor recurrence is a major complication after hepatectomy and a leading cause of cancer-related death. It is usually divided into early recurrence and late recurrence by 2 years (5, 6). Previous studies have shown that the early recurrence rate of HCC is as high as 30-50%, accounting for more than 70% of the total tumor recurrence (6–8). Timely identification of high-risk patients with early recurrence after surgery is very important for prolonging the survival time of patients and improving the quality of life after surgery. At present, there are many predictive models established in different medical centers based on risk factors related to early recurrence (such as male, large Tumor diameter, high serum AFP, vascular invasion, low tumor differentiation, etc.) and imaging features (9, 10). However, there is no consensus on the best tool for risk stratification.

The construction of clinical prediction models based on inflammation-related indicators is a research hotspot in recent years. Since the 20th century, the theory of cancer-related inflammation has been enriched and developed, and the role of inflammation in tumorigenesis, proliferation, invasion, and metastasis has been gradually elucidated (11, 12). Inflammatory indexes constructed based on peripheral blood neutrophil, lymphocyte, monocyte and platelet counts have been developed for cancer research due to their non-invasive, clinically readily available and low-cost nature. In a variety of malignant tumors, these inflammatory indexes have a good effect in predicting prognosis (13–15). In HCC, these inflammatory indicators such as neutrophil-to-lymphocyte ratio (NLR), platelet-to-lymphocyte ratio (PLR), systemic inflammatory response index (SIRI) and systemic immune inflammation index (SII) have also shown high application value in predicting HCC prognosis (16–18). However, previous studies only included 1-2 inflammatory markers. There is still a paucity of literature that combines these 4 important inflammatory markers to study early recurrence of HBV-related HCC after surgery. In this study, we compared the effects of four inflammatory markers models on the early recurrence of HBV-related HCC after surgery in the same population and constructed a common prediction model based on inflammatory markers and pathological characteristics. By comparing the predictive efficacy of each model, we found a more robust and accurate model that could predict the early recurrence of HBV-related HCC after surgery.

## 2 Materials and methods

### 2.1 Patient and clinical sample collection

We investigated 162 patients with HBV-related HCC who underwent hepatectomy at Shanxi Provincial Cancer Hospital from January 2013 to April 2016. Clinical and demographic data and detailed treatment information for all enrolled patients were extracted from the electronic medical record. The inclusion criteria were as follows: (1) HCC was diagnosed by postoperative pathology; (2) negative surgical margins; (3) liver reserve function Child-Pugh grade A or B; (4) patients over 18 years old; (5) complete clinical and pathological data were available. Exclusion criteria: (1) acute infection or high fever before surgery; (2) other malignant tumors, immune or hematological diseases; (3) preoperative anti-tumor therapy; (4) loss of critical data. This retrospective chart review study involving human participants complied with institutional and national research Council ethical standards and the 1964 Declaration of Helsinki.

The baseline clinical data of the patients were collected and analyzed, including gender, age, body mass index (BMI), diabetes mellitus, drinking history, family history of cancer, hepatitis B surface antigen (HBsAg), AJCC stage, tumor number, tumor diameter, tumor differentiation, vascular invasion, liver cirrhosis, postoperative treatment (including postoperative ablation or TACE), serum alpha-fetoprotein (AFP) and preoperative blood routine test. Routine blood samples were collected within 7 days before surgery. AJCC staging was performed according to the 8th edition of the classification of the Union for International Cancer Control and the American Joint Committee on Cancer (AJCC) (19).

Peripheral blood inflammation-related indicators refer to relevant indicators that can reflect systemic inflammation based on immune cells of the human circulatory system (13–18). The formula for the calculation of various inflammation-related indicators based on blood routine test was as follows: SIRI = neutrophil×monocyte/lymphocyte (13); PLR = platelet/lymphocyte (18); NLR= neutrophil/lymphocyte (18), SII = platelet ×neutrophil/lymphocyte (17).

### 2.2 Follow-up

Follow-up began after surgery and was performed every 3 months in the outpatient clinic for the first 2 years until recurrence or loss of follow-up. During follow-up, examinations included physical examination, AFP testing, and abdominal imaging scans. If there was no evidence of recurrence, chest X-ray and abdominal ultrasound were preferred. If abdominal ultrasound suggested recurrence, abdominal enhanced CT scan or magnetic resonance scan should be performed to confirm the diagnosis. In addition, chest CT enhancement should be used to rule out lung metastases, and positron emission tomography should be used to rule out metastases in other sites. Outpatient and inpatient medical record systems were combined with telephone follow-up. Recurrence-free survival (RFS) was defined as the time from the date of surgery to the first recurrence or loss to follow-up.

### 2.3 Statistical analysis

Taking the recurrence within 2 years as the outcome, ROC analysis was performed on the original values of inflammatory markers as variables, and the best cut-off value was found by the Youden index. The population was divided into high and low groups by cut-off value. The Pearson Chi-square test was used to evaluate the association between SIRI, PLR, NLR, SII and clinical and pathological data. The Kaplan-Meier method and Log-Rank test were used to analyze the differences between groups and recurrence-free survival rate within 2 years. The Cox regression analysis was used to identify the risk factors for early postoperative recurrence. According to the results, the regression coefficients of the included variables were obtained by binary logistic regression equation, and the joint predictors were obtained by the regression coefficients. The SPSS 24.0 and GraphPad Prism 9 were used for statistical analysis and drawing. The MedCalc(20.218) was used to conduct DeLong’s test. P<0.05 considered that the difference was statistically significant.

## 3 Results

### 3.1 Baseline data

A total of 162 HCC patients who underwent partial hepatectomy were enrolled. 58 patients had recurrence within 2 years, including 37 cases of intrahepatic recurrence, 7 cases of extrahepatic recurrence and 14 cases of both intrahepatic and extrahepatic recurrence, with a recurrence rate of 35.8%. ROC analysis showed that the optimal cut-off points of SIRI, PLR, NLR and SII were 0.785, 86.421, 2.231 and 353.64, respectively. HCC patients were divided into low SIRI group (≤0.785) and high SIRI group (>0.785), low PLR group (≤86.421), high PLR group (>86.421), low NLR group (≤2.231), high NLR group (>2.231), low SII group (≤353.64) and high SII group (>353.64). **Table 1** detailed the association of different groups with the clinical and pathological features. Of the 162 patients, 125 (77.2%) were male and 37 (22.8%) were female. 107 cases (66.0%) were under 60 years, and 55 cases (34.0%) were over 60 years. There were 93 patients (57.4%) in the low SIRI group and 69 patients (42.6%) in the high SIRI group. There were 74 patients (45.7%) in the low PLR group and 88 patients (54.3%) in the high PLR group. There were 98 patients (60.5%) in the low NLR group and 69 patients (39.5%) in the high NLR group. There were 93 patients (57.4%) in the low SII group and 64 patients (42.6%) in the high SII group. The results showed that SIRI, PLR, NLR and SII were significantly correlated with tumor diameter (*P*< 0.05). PLR was significantly different in age and liver cirrhosis (*P*<0.05). NLR was associated with the type of tumor differentiation and the use of TACE/ablation after surgery (*P*< 0.05). SII had a statistically significant difference in HBsAg (+) (*P*< 0.05).

**Table 1:** Baseline Characteristics of 162 Patients with HCC.

Variables	No.(%)	SIRI		χ²	P value	PLR		χ²	P value	NLR		χ²	P value	SII		χ²		P value	
		≼	>			≼	>			≼2.231	>2.231			≼353.64	>353.64				
		0.785	0.785			86.421	86.421												
All patients	162(100.0)	93	69			74	88			98	64			93	69			
Gender																			
Male	125(77.2)	71	54	0.083	0.774	61	64	2.148	0.143	78	47	0.832	0.362	73	52	0.221		0.639	
Female	37(22.8)	22	15			13	24			20	17			20	17				
Age																			
≤60	107(66.0)	64	43	0.746	0.388	55	52	4.160	0.041	65	42	0.008	0.927	63	44	0.279		0.597	
>60	55(34.0)	29	26			19	36			33	22			30	25				
BMI																			
≼28.0	150(92.5)	80	63	1.068	0.301	67	76	0.677	0.410	86	57	0.064	0.800	84	59	0.887		0.346	
>28.0	12(7.5)	13	6			7	12			12	7			9	10				
Diabetes																			
No	151(93.2)	86	65	0.187	0.665	70	81	0.413	0.521	91	60	0.049	0.825	87	64	0.040		0.842	
Yes	11(6.8)	7	4			4	7			7	4			6	5				
Alcohol																			
No	130(80.2)	74	56	0.063	0.802	60	70	0.060	0.807	79	51	0.021	0.885	76	54	0.299		0.584	
Yes	32(19.8)	19	13			14	18			19	13			17	15				
Family cancer history																			
No	150(92.5)	86	66	0.691	0.406	59	83	0.080	0.777	92	60	0.001	0.974	89	63	1.321		0.250	
Yes	12(7.5)	7	3			5	5			6	4			4	6				
HBsAg (+)																			
No	27(16.7)	15	12	0.045	0.831	8	19	3.363	0.067	15	12	0.331	0.565	9	18	7.680		0.006	
Yes	135(83.3)	78	57			66	69			83	52			83	51				
Tumor number																			
Multiple	24(14.8)	15	9	0.299	0.585	11	13	0.000	0.987	16	8	0.449	0.503	16	8	0.988		0.320	
Solitary	138(85.2)	78	60			63	75			82	56			77	61				
Tumor diameter																			
≼5	101(62.3)	69	32	13.056	0.000	55	46	8.326	0.004	69	32	6.869	0.009	70	31	15.533		0.000	
>5	61(37.7)	24	37			19	42			29	32			23	38				
Differentiation																			
poor	60(37.0)	30	30	2.138	0.144	23	37	2.072	0.150	27	33	9.572	0.002	30	30	2.138		0.144	
Moderate and well	102(63.0)	63	39			51	51			71	31			63	39				
Vascular invasion																			
No	147(90.7)	87	60	2.049	0.152	70	77	2.408	0.121	91	56	1.322	0.250	86	61	0.780		0.377	
Yes	15(9.3)	6	9			4	11			7	8			7	8				
Liver cirrhosis																			
No	26(16.0)	14	12	0.161	0.689	6	20	6.376	0.012	16	10	0.014	0.905	13	13	0.695		0.404	
Yes	136(84.0)	79	57			68	68			82	54			80	56				
N																			
No	157(97.0)	90	67	0.014	0.905	73	84	1.371	0.242	95	62	0.001	0.982	91	66	0.639		0.424	
Yes	5(3.0)	3	2			1	4			3	2			2	3				
AJCC stage																			
I-II	154(95.0)	88	66	0.089	0.765	72	82	1.450	0.228	92	62	0.741	0.389	88	66	0.089		0.765	
III-IV	8(5.0)	5	3			2	6			6	2			5	3				
Postoperative treatment (Ablation or TACE)																			
No	135(83.3)	78	57	0.045	0.831	64	71	0.975	0.323	88	47	7.459	0.006	81	54	2.227		0.136	
Yes	27(16.7)	15	12			10	17			10	17			12	15				
AFP>400																			
No	112(69.1)	65	47	0.059	0.809	70	42	0.611	0.434	65	47	0.059	0.809	73	39	0.152		0.697	
Yes	50(30.9)	28	22			28	22			28	22			31	19				
Child-Pugh grading																			
A	154(95.0)	89	65	0.189	0.664	72	82	1.450	0.228	94	60	0.388	0.533	90	64	1.364		0.240	
B	8(5.0)	4	4			2	6			4	4			3	5				

SIRI, Systemic Inflammation Response Index; PLR, Platelet-to-lymphocyte ratio; NLR, Neutrophil-to-Lymphocyte Ratio; SII, Systemic Immune-Inflammation Index; BMI, Body mass index; N, Regional lymph node metastasis; AFP, alpha fetoprotein; TACE, transcatheter arterial chemoembolization.

### 3.2 Differences in RFS between groups

**Figure 1:**
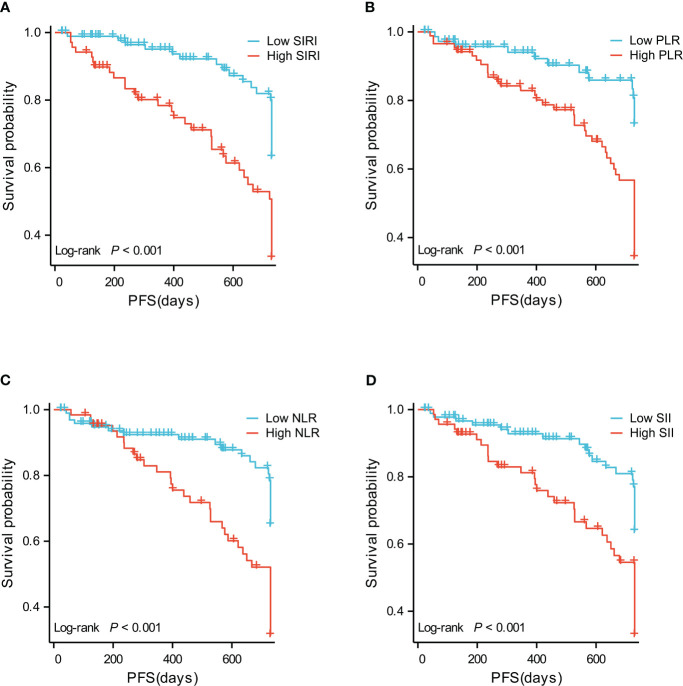
**(A)** Recurrence-free survival curve of patients with high SIRI and low SIRI. **(B)** Recurrence-free survival curve of patients with high PLR and low PLR. **(C)** Recurrence-free survival curve of patients with high NLR and low NLR. **(D)** Recurrence-free survival curves of patients with high SII and low SII.

The difference between SIRI ≤ 0.785 group and SIRI>0.785 groups was tested by Log-Rank test, and P<0.001 (**Figure 1A**), indicating that there was a significant difference between groups in terms of recurrence-free survival rate. The 2-year recurrence-free survival rate of the SIRI ≤ 0.785 group was 76.3%, the other was 47.8%.

The difference between PLR ≤ 86.421 group and PLR>86.421 group was tested by Log-Rank test, and P<0.001 (**Figure 1B**), indicating that there was a significant difference between groups in terms of recurrence-free survival rate. The 2-year recurrence-free survival rate of the PLR ≤ 86.421 group was 82.7%, the other was 48.2%.

The difference between NLR ≤ 2.231 group and NLR>2.231 group was tested by Log-Rank test, and P<0.001 (**Figure 1C**), indicating that there was a significant difference between groups in terms of recurrence-free survival rate. The 2-year recurrence-free survival rate of the NLR ≤ 2.231 group was 77.8%, the other was 42.9%.

The difference between SII ≤ 353.64 group and SII>353.64 group was tested by Log-Rank test, and P<0.001 (**Figure 1D**), indicating that there was a significant difference between groups in terms of recurrence-free survival rate. The 2-year recurrence-free survival rate of the SII ≤ 353.64 group was 76.3%, the other was 47.8%.

### 3.3 Univariate and multivariate COX regression analysis

Cox univariate analysis of tumor diameter (>5cm vs ≤5cm: *HR*=1.700, 95%*CI*: 1.015-2.845, *P*=0.044), degree of differentiation (poorly differentiated vs moderately or well differentiated: *HR*=2.438, 95%*CI*: 1.449-4.103, *P*<0.001), vascular invasion (invasion vs no invasion: *HR*=3.053, 95%*CI*: 1.519-6.136, *P*=0.002), SIRI (>0.785 vs ≤0.785: *HR*=2.738, 95%*CI*:1.609-4.660, *P*<0.001), PLR(>86.421 vs ≤86.421, *HR*=3.060, 95%*CI*: 1.649-5.676, *P*< 0.001), NLR(>2.231 vs ≤2.231, *HR*=2.613, 95%*CI*: 1.536-4.443, *P*< 0.001), SII(>353.64 vs ≤353.64, *HR*=2.547, 95%*CI*: 1.497-4.334, *P*<0.001) (**Table 2**). Multivariate analysis showed that only tumor differentiation (poor differentiation vs moderate-high differentiation: *HR*=2.043, 95%*CI*: 1.152-3.623, *P*=0.014) was an independent risk factor for early recurrence within 2 years (**Table 2**).

**Table 2:** Univariate and multivariate analysis of RFS within 2 years.

Characteristics	Total (N)	Univariate analysis		Multivariate analysis	
		Hazard ratio (95% CI)	P value	Hazard ratio (95% CI)	P value
Gender	162				
Female	37	Reference			
Male	125	1.154 (0.611-2.179)	0.658		
Age	162				
≤60	107	Reference			
>60	55	1.104 (0.642-1.898)	0.720		
BMI>28	162				
≤28	143	Reference			
>28	19	1.848 (0.873-3.916)	0.109		
Diabetes	162				
No	151	Reference			
Yes	11	0.485 (0.151-1.552)	0.222		
Drinking history	162				
No	130	Reference			
Yes	32	1.432 (0.795-2.578)	0.232		
HBSAg(+)	162				
No	27	Reference			
Yes	135	0.748 (0.387-1.443)	0.386		
Tumor number	162				
Solitary	138	Reference			
Multiple	24	0.887 (0.420-1.872)	0.754		
Tumor diameter (5cm)	162				
≤5	101	Reference			
>5	61	1.700 (1.015-2.845)	0.044	1.338 (0.763-2.343)	0.309
Differentiation	162				
Moderate and well	102	Reference			
poor	60	2.438 (1.449-4.103)	<0.001	2.043 (1.152-3.623)	0.014
Vascular invasion	162				
No	147	Reference			
Yes	15	3.053 (1.519-6.136)	0.002	1.959 (0.936-4.097)	0.074
Cirrhosis	162				
No	26	Reference			
Yes	136	0.574 (0.304-1.085)	0.087		
AJCC Staging	162				
I-II	154	Reference			
III-IV	8	1.609 (0.581-4.456)	0.360		
Postoperative treatment (Ablation or TACE)	162				
No	135	Reference			
Yes	27	1.274 (0.687-2.364)	0.442		
SIRI					
≤0.785	93	Reference			
>0.785	69	2.738 (1.609-4.660)	<0.001	1.690 (0.848-3.368)	0.136
PLR	162				
≤86.421	74	Reference			
>86.421	88	3.060 (1.649-5.676)	<0.001	1.758 (0.859-3.600)	0.123
NLR	162				
≤2.231	98	Reference			
>2.231	64	2.613 (1.536-4.443)	<0.001	1.113 (0.554-2.238)	0.763
SII	162				
≤353.64	93	Reference			
>353.64	69	2.547 (1.497-4.334)	<0.001	1.319 (0.674-2.581)	0.419
AFP (ng/ml)	162				
≤400	112	Reference			
>400	50	1.124 (0.649-1.946)	0.676		

SIRI, Systemic Inflammation Response Index; PLR, Platelet-to-Lymphocyte Ratio; NLR, Neutrophil-to-Lymphocyte Ratio; SII, Systemic Immune-Inflammation Index; BMI, Body mass index; N, Regional lymph node metastasis; AFP, alpha fetoprotein.

### 3.4 Joint index construction

**Figure 2:**
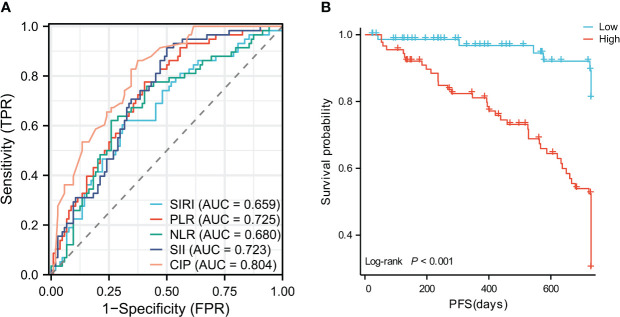
**(A)** Receiver operating characteristic analysis of SIRI, PLR, NLR, SII,CIP. **(B)** Recurrence-free survival curves of patients with high CIP and low CIP.

According to the results of univariate and multivariate analysis, based on the available clinical and pathological parameters, the 4 inflammatory indicators combined with tumor diameter, degree of differentiation and vascular invasion were used to construct a combined inflammation and pathology model, referred to as CIP. Scoring criteria: tumor diameter (>5cm=1,≤5cm=0), degree of differentiation (undifferentiated and poorly differentiated=1, moderately and well differentiated=0), vascular invasion (with invasion=1, no invasion=0), SIRI (>0.785 = 1,≤0.785 = 0), PLR (>86.421 = 1,≤86.421 = 0), NLR(>2.231 = 1,≤2.231 = 0), SII(>353.64 = 1,≤353.64 = 0). According to the regression coefficient of binary logistic equation, the joint index calculation formula was as follows: CIP=0.331×Tumor diameter +1.141×degree of differentiation +0.970×vascular invasion+0.469×SIRI+1.152×PLR+0.630×NLR+0.031×SII. The area under the ROC curve of joint index CIP was 0.804 (the best cut-off value was 1.48), which was higher than that of other single indexes (SIRI=0.659, PLR=0.725, NLR=0.680, SII=0.723) (**Figure 2A**). DeLong’s test was performed using MedCalc (20.218), and it was found that there was no significant difference between the single inflammation indexes, and the joint index CIP was significantly different from the single inflammation models (*P*<0.05) (**Table 3**). The KM curve showed that the group with a lower joint index had a longer RFS (P<0.001) (**Figure 2B**). To avoid the interaction between variables, univariate and multivariate cox analysis were performed again after removing the factors included in the combined index. The joint index was an independent risk factor for early recurrence and had good predictive value (**Table 4**).

**Table 3:** DeLong’s test between models.

Model 1	Model 2	P value
SIRI	PLR	0.191
SIRI	NLR	0.5528
SIRI	SII	0.062
SIRI	CIP	0.0006
PLR	NLR	0.3621
PLR	SII	0.9536
PLR	CIP	0.0115
NLR	SII	0.2426
NLR	CIP	0.0012
SII	CIP	0.0091

SIRI, Systemic Inflammation Response Index; PLR, Platelet-to-lymphocyte ratio; NLR, Neutrophil-to-Lymphocyte Ratio; SII, Systemic Immune-Inflammation Index; CIP, combined inflammation and pathology index.

**Table 4:** Univariate and multivariate analysis of RFS within 2 years.

Characteristics	Total (N)	Univariate analysis		Multivariate analysis	
		Hazard ratio (95% CI)	P value	Hazard ratio (95% CI)	P value
Gender	162				
Female	37	Reference			
Male	125	1.154 (0.611-2.179)	0.658		
Age	162				
≤60	107	Reference			
>60	55	1.104 (0.642-1.898)	0.720		
BMI>28	162				
≤28	143	Reference			
>28	19	1.848 (0.873-3.916)	0.109		
Diabetes	162				
No	151	Reference			
Yes	11	0.485 (0.151-1.552)	0.222		
Drinking history	162				
No	130	Reference			
Yes	32	1.432 (0.795-2.578)	0.232		
HBSAg (+)	162				
No	27	Reference			
Yes	135	0.748 (0.387-1.443)	0.386		
Tumor number	162				
Solitary	138	Reference			
Multiple	24	0.887 (0.420-1.872)	0.754		
Cirrhosis	162				
No	26	Reference			
Yes	136	0.574 (0.304-1.085)	0.087		
AJCC Staging	162				
I-II	154	Reference			
III-IV	8	1.609 (0.581-4.456)	0.360		
Postoperative treatment (Ablation or TACE)	162				
No	135	Reference			
Yes	27	1.274 (0.687-2.364)	0.442		
AFP (ng/ml)	162				
≤400	112	Reference			
>400	50	1.124 (0.649-1.946)	0.676		
CIP					
≤1.48	73	Reference			
>1.48	89	5.851 (2.770 - 12.360)	< 0.001	5.851 (2.770 - 12.360)	< 0.001

SIRI, Systemic Inflammation Response Index; PLR, Platelet-to-Lymphocyte Ratio; NLR, Neutrophil-to-Lymphocyte Ratio; SII, Systemic Immune-Inflammation Index; BMI, Body mass index; N, Regional lymph node metastasis; AFP, alpha fetoprotein.

## 4 Discussion

Cancer-associated inflammation can be divided into two categories: local inflammation and systemic inflammation. Local inflammation is mainly related to the immune response in the tumor microenvironment, which usually occurs before the appearance of tumors. Systemic inflammation is a continuous response to malignant tumors mediated by cytokines, inflammatory proteins, and immune cells (20). HBV-related HCC is an inflammation-driven tumor, which mostly occurs based on chronic hepatitis, and its development, proliferation and metastasis are seriously affected by the inflammatory environment (21). Currently, there is no consensus on the time point of early recurrence, which ranges from 6 months to 2 years in most studies (22–24). It is generally accepted that recurrences within 2 years represent “true recurrences,” whereas after this period, “recurrences” are thought to be largely caused by “*de novo*” tumors (6). Our study used 2 years as the cutoff point. Whether 2 years is the best cut-off point to determine early recurrence needs further study.

There have been many studies on the effects of inflammatory indexes on RFS and overall survival of HCC after surgery. However, there are few studies on the effect of inflammatory indexes on early recurrence (within 2 years) of liver cancer after surgery. In 2021, Wu et al. proposed that inflammatory indexes could be combined with clinical risk factors to construct a more effective prediction model for early recurrence (25). Our study focused on patients with HBV-related HCC, explored the predictive value of SIRI, PLR, NLR, and SII for early recurrence, and constructed a joint index CIP combining inflammatory indicators and pathological features. Compared with the model constructed by single inflammatory indexes, the CIP model had better predictive ability (AUC=0.804). It is worthy of further research and promotion.

Studies showed that all 4 inflammatory indexes were associated with tumor diameter and high SIRI, PLR, NLR and SII were associated with shorter RFS within 2 years. This may be due to the role of immune cells that constitute the inflammatory markers. Elevated levels of circulating neutrophils have been linked to the stimulation of tumor-derived cytokines, such as granulocyte colony-stimulating factor, platelet-derived growth factor, and Interleukin-8, which mobilize bone marrow-derived cells and splenocytes and lead to their circulation and migration to organs (26). Neutrophils are thought to drive tumor progression through immunosuppression, direct enhancement of tumor cell survival, invasiveness, and metastatic ability, extracellular matrix remodeling, and angiogenesis (27). Lymphocytes control tumor growth by inducing cytotoxic cell death and secreting cytokines, and decreased levels of lymphocytes can impair host immune function and accelerate tumor progression (28). Monocytes can infiltrate tumors and further differentiate into Tumor-associated macrophages, which can induce apoptosis of CD8+ T cells with anticancer activity and promote tumor growth, invasion, and migration (29). It has been suggested that macrophage populations, as indicated by peripheral blood mononuclear cell counts, are correlated with tumor burden. In this study, it was found that platelet-related parameters such as PLR and SII had better predictive performance. It has been proposed that platelets can promote tumor growth and metastasis through the release of mediators such as vascular endothelial growth factor and platelet-derived growth factor. Additionally, platelets may also protect tumor cells from natural killer cells and promote epithelial-mesenchymal transition. It is suggested that high platelet counts may be associated with a poor prognosis in HCC.

Tumor markers such as alpha-fetoprotein and abnormal prothrombin are effective indicators to judge the prognosis of HCC, but many patients have normal tumor markers when they are diagnosed with HCC. Therefore, it is essential to find more prognostic biomarkers for clinical decision-making. Peripheral blood inflammatory markers are highly generalizable due to their non-invasive and easy availability. Future prospective multicenter studies with larger sample sizes and studies targeting other HCC etiologies are necessary to verify the results of this study.

## 5 Conclusion

The joint index CIP constructed by combining preoperative SIRI, PLR, NLP and SII with pathological features, can better predict the early recurrence of HBV-related HCC patients after surgery, which is helpful to identify high-risk patients and assist clinicians to make better clinical decisions.

## Data availability statement

The datasets presented in this article are not readily available because hepatocellular carcinoma. Requests to access the datasets should be directed to 953168503@qq.com

## Ethics statement

The studies involving human participants were reviewed and approved by Shanxi Provincial Cancer Hospital. Written informed consent for participation was not required for this study in accordance with the national legislation and the institutional requirements.

## Author contributions

GW is the main writer. LY, MJ and WB collected the data. LY also organized the data and participated in writing the paper. LLB analyzed the data. NZ and NY reviewed and revised the manuscript. LLX designed the overall topic and guiding the paper. All the authors contributed to the original draft preparation and reviewing and editing of the study.
